# Receptor-interacting protein 140 as a co-repressor of Heat Shock Factor 1 regulates neuronal stress response

**DOI:** 10.1038/s41419-017-0008-5

**Published:** 2017-12-12

**Authors:** Yu-Lung Lin, Hong-Chieh Tsai, Pei-Yao Liu, Michael Benneyworth, Li-Na Wei

**Affiliations:** 1 Department of Pharmacology, University of Minnesota, Minneapolis, MN 55455 USA; 2 Graduate Institute of Clinical Medical Sciences, College of Medicine, Chang-Gung University, Tao-Yuan, Taiwan, ROC; 3 Department of Neurosurgery, Chang-Gung Memorial Hospital and University, Tao-Yuan, Taiwan, ROC; 4 Departments of Neuroscience, University of Minnesota, Minneapolis, MN 55455 USA

## Abstract

Heat shock response (HSR) is a highly conserved transcriptional program that protects organisms against various stressful conditions. However, the molecular mechanisms modulating HSR, especially the suppression of HSR, is poorly understood. Here, we found that RIP140, a wide-spectrum cofactor of nuclear hormone receptors, acts as a co-repressor of heat shock factor 1 (HSF1) to suppress HSR in healthy neurons. When neurons are stressed such as by heat shock or sodium arsenite (As), cells engage specific proteosome-mediated degradation to reduce RIP140 level, thereby relieving the suppression and activating HSR. RIP140 degradation requires specific Tyr-phosphorylation by Syk that is activated in stressful conditions. Lowering RIP140 level protects hippocampal neurons from As stress, significantly it increases neuron survival and improves spine density. Reducing hippocampal RIP140 in the mouse rescues chronic As-induced spatial learning deficits. This is the first study elucidating RIP140-mediated suppression of HSF1-activated HSR in neurons and brain. Importantly, degradation of RIP140 in stressed neurons relieves this suppression, allowing neurons to efficiently and timely engage HSR programs and recover. Therefore, stimulating RIP140 degradation to activate anti-stress program provides a potential preventive or therapeutic strategy for neurodegeneration diseases.

## Introduction

Heat shock response (HSR) is a highly conserved cellular process that protects cells from diverse environmental and physiological stressors, such as heat shock (HS), heavy metals, oxidative stress, endoplasmic reticulum (ER) stress, infections and cancers etc.^1–4^ These stressors induce the expression of heat shock proteins (HSPs) including molecular chaperones and proteases that are important for protection and recovery from cellular stress. Heat shock factor 1 (HSF1) is the master transcription factor that activates genes during HSR. In unstressed cells, HSF1 is maintained in an inactive monomeric form by forming a complex with HSP90 and HSP70 in the cytoplasm. During stress, HSF1 undergoes a multi-step activation process to dissociate from the chaperone complex, translocate into the nucleus, and form active phosphorylated trimers. The activated HSF1 binds to the heat shock element (HSE) of target genes, such as genes for HSP70, HSP90 and other chaperone family proteins, to activate their transcription.^5^ Studies have shown HSF1 activity regulated by its post-translational modifications^6,7^ and, critically, coactivators such as ASC-2, Strap, p300, and MLL1.^8–10^ Interestingly, two corepressors of HSF-1 have been reported, i.e., PGC-1α for repressing HSF1 in metabolic tissues such as liver and muscle during fasting^11^, and CoREST, which was demonstrated using overexpression in HEK293 cell line.^12^ However, the physiological context where HSF1 represses, instead of activates, gene expression remains to be further examined. In particular, whether and how this plays out in the nervous system is still unclear.

Arsenic is ubiquitously present in the environment. Inorganic arsenic is among the top of hazardous substances^13^ and one of WHO’s ten chemicals of major public health concerns.^14^ Arsenic toxicity is related to cancers,^15^ developmental neurotoxicity, and central nervous system (CNS) dysfunctions.^16^ Epidemiology studies indicate that long-term exposure to arsenic contamination from drinking-water decreases cognitive functions and increases the risk of psychiatric disorders in human^17^ because arsenic can easily penetrate the blood–brain barrier and accumulate in the brain, especially the hippocampus.^18,19^ Acute or chronic exposure to sodium arsenite (As) results in hippocampus-dependent behavioral changes associated with deficits in learning and memory.^17,20,21^ The most widely accepted mechanism of As toxicity is through the generation of reactive oxygen species (ROS).^22^ As, or its monomethylated metabolite, also affects protein homeostasis and induces ER stress.^23–25^ As-stressed cells respond by activating HSR;^26,27^ however, how HSR is modulated to timely protect As-induced damage in the nervous system is unclear.

Receptor-interacting protein 140 (RIP140) is a wide-spectrum transcriptional co-regulator that functions in various organ systems and biological processes including metabolism, development, and inflammation.^28–32^ Its biological activity is attributed to, mainly, its acting as a transcription co-repressor that recruits a wide variety of repressive transcription cofactors such as histone deacetylases (HDACs),^33,34^ chromatin remodeler Brm,^35^ lysine demethylase LSD1,^36^ and histone methyl transferase G9a^37^ etc. Recently, we discovered that RIP140 is involved in cellular responses to environmental and physiological stressors. For instance, ER stress such as Aβ induces a fraction of RIP140 translocation to the cytosol to modulate IP_3_R activity and attenuate uncontrolled Ca^2+^ release in neuron.^38^ Further, sub-chronic psychological stress reduces RIP140 protein expression in astrocyte and disrupts brain cholesterol homeostasi.s^39,40^ Importantly, RIP140 expression is enriched in cortex and hippocampus, areas associated with Alzheimer’s disease (AD) pathology, and its expression is lower in AD postmortem brains.^41^ All these observations suggest that RIP140 can be a stress modulator in the brain. However, it is unclear whether and how RIP140 may be involved in modulating HSR in the brain.

In the current study, we describe that in neurons and the brain, particularly the hippocampus, RIP140 acts as a transcriptional co-repressor of HSF1. This serves to suppress the unwanted expression of HSP genes in healthy, unstressed neurons. In stressed neurons, such as those under HS or exposed to As, RIP140 protein level is rapidly reduced by proteasome-mediated protein degradation. This rapid drop in RIP140 protein level relieves the suppression of HSF1 target genes, allowing HSP gene transcription timely elevated for cell survival. We also demonstrate that mice exposed to chronic As treatment are compromised in their hippocampus-dependent learning, and this deficit can be rescued by first silencing RIP140 expression (to elevate HSR) in the hippocampus prior to As challenges. This current study reveals a novel RIP140-mediated neuronal stress modulatory mechanism, which involves rapid protein quality control of RIP140 that is a co-repressor of HSF1.

## Results

### RIP140 suppresses HSF1 target genes, and stress triggers loss of RIP140 protein in hippocampal neurons

**Fig. 1:**
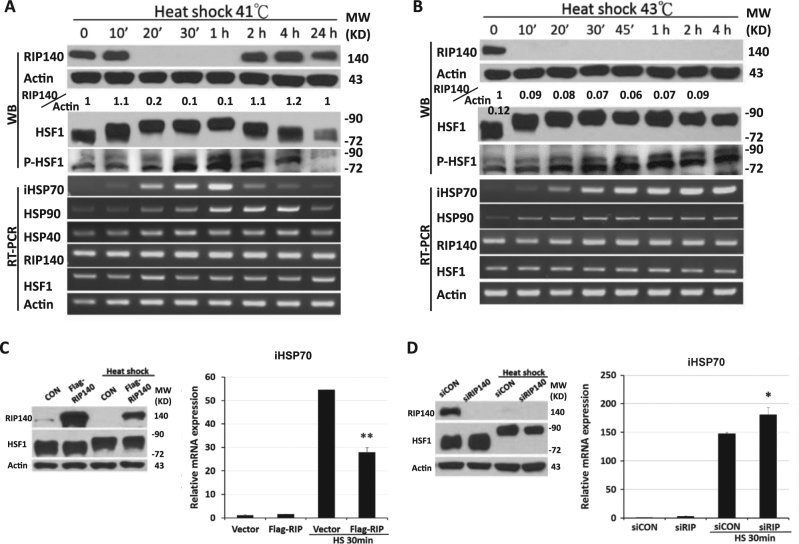
Heat shock-triggered RIP140 disappearance enhances HSR **a**, **b** Expression of RIP140, HSF1, P-HSF1, and HSPs in hippocampal neural cell line HT22 stressed at 41 or 43 °C. Quantified and normalized expression of RIP140 is shown under the corresponding blots. **c** Effects of RIP140 overexpression on iHSP70 mRNA expression at 41 °C (*N* = 3). **d** Effects of RIP140 knockdown on iHSP70 mRNA expression at 41 °C (*N* = 3). Results are presented as means ± SEM, *p < 0.05, **p < 0.01 compared with HS control group

To examine the relationship between RIP140 and HSR, we first employed a hippocampal neural cell line HT22 to examine responses to HS at 41 °C. Figure 1a shows that HS very effectively and rapidly (20 min) induces HSF1 activation (indicated by bend shift that is caused by post-translational modifications) and messenger RNA (mRNA) expression of HSPs, which subsides at approximately 2 h as cells recover from stress. Interesting, RIP140 also disappears at 20 min and reappears at approximately 2 h after HS. At a further elevated temperature such as 43 °C where HSR is detected earlier (at 10 min) and lasts for the entire duration of examination (4 h), RIP140 also disappears beginning at 10 min, and through out the entire time of examination (Fig. 1b). These results indicate a close relationship between the disappearance of RIP140 and the activation of HSR.

To determine the functional role of RIP140 in HSR, we carried out gain- and loss-of-function studies using the same experimental system. As shown in Fig. 1c, overexpressing RIP140 effectively suppresses the activation of inducible HSP70 (iHSP70; also known as HSP72, HSP70-1, and HSPa1a), a major HSF1 target. On the contrary, silencing RIP140 expression elevates the level of iHSP70 (Fig. 1d).

**Fig. 2:**
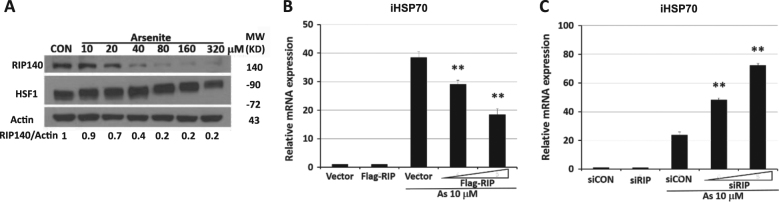
As-induced RIP140 disappearance enhances HSR **a** Changes in RIP140 levels in HT22 cells stressed with As at various doses. Quantified and normalized expression level of RIP140 is shown under the corresponding blots. **b** Effects of RIP140 overexpression on iHSP70 mRNA expression in cells stressed with 10 μM As for 1 h (*N* = 3). **c** Effects of RIP140 knockdown on iHSP70 mRNA expression in cells stressed with 10 μM As for 1 h (*N* = 3). Results are presented as means ± SEM, **p < 0.01 compared with As-stressed control

To further examine if this represents a general phenomenon related to HSR, we employed As to induce oxidative stress that also activates HSF1-mediated HSR.^22,26,27^ Interestingly, As treatment also reduces RIP140 level, but it occurs in a dose-dependent manner. As at concentrations higher than 40 μM very effectively reduces RIP140 levels within 1 h, whereas As at a lower concentration (such as 10 μM) only triggers negligible reduction in RIP140 (Fig. 2a). We thus applied 10 μM As to evaluate the functional role of RIP140 in gain-of-function studies, because cells under this low stress level retain their RIP140. As shown in Fig. 2b, in cells stressed with 10 μM As, overexpressing RIP140 causes a 50% decrease in iHSP70 expression in a RIP140 dose-dependent manner; whereas silencing RIP140 results in more than threefold increases in the induction of iHSP70 (Fig. 2c).

Results using these stress models consistently show that RIP140 functions as a suppressor of HSR, likely by co-repressing HSF1 targets. Further, RIP140 is rapidly lost in cells exposed to stressors such as HS (41 °C for 20 min or 43 °C for 10 min) or high concentrations of As (>40 μM). Presumably, in stressed cells, losing RIP140 could relieve this suppression, allowing cells to rapidly activate the otherwise suppressed HSR and HSF1 to facilitate recovery from stress and cell survival.

### Reducing RIP140 enhances HSR in primary neurons and protects mice from As-induced hippocampus-dependent learning deficits

**Fig. 3:**
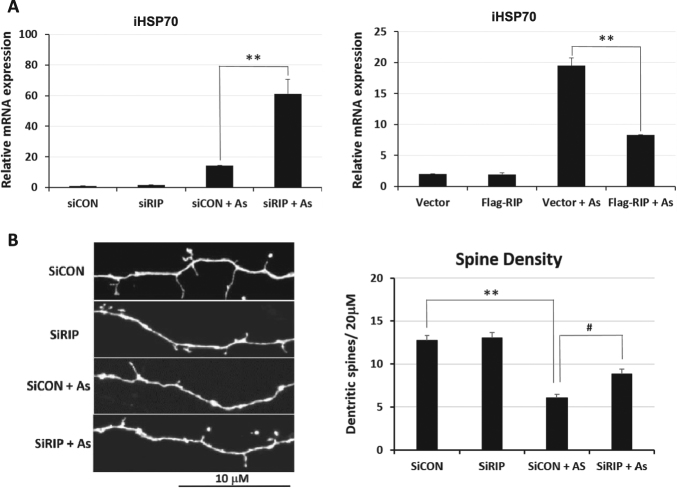
Silencing RIP140 enhances HSR and protects hippocampal neurons from As-induced oxidative stress **a** Effects of RIP140 overexpression and knockdown on HSP70 mRNA expression in primary hippocampal neurons stressed with 10 μM As for 1 h (*N* = 3). **b** Confocal image and spine quantification showing rescue of As-damaged dendritic spine by silencing RIP140 (*N* = 10 neurons). Original sections where these images were acquired are shown in Supplementary Fig. 1. Results are presented as means ± SEM, *^#^ *p* < 0.05, **p < 0.01 comparing the respective groups

To determine whether RIP140 acts to modulate HSR in vivo, we examined both primary hippocampal neuron cultures and mice exposed to As-induced oxidative stress. As shown in Fig. 3a, the induction of iHSP70 in primary hippocampal neurons exposed to 2 μM As for 1 h is further elevated by silencing RIP140 (left), but is significantly dampened by overexpressing RIP140 (right). To evaluate whether reducing RIP140 (to enhance HSR) can indeed protect As-triggered neuronal damage, we monitored their spine density. As shown in Fig. 3b (original sections shown in Supplementary Fig. 1), in unstressed neurons, silencing RIP140 has no effect on spine density, indicating that in normal healthy neurons the level of RIP140 does not impact on cell survival within the time window examined. As stress significantly reduces spine density as expected. Interestingly, As-induced spine damage is effectively rescued by first silencing RIP140 (elevating HSR) in these neurons. In fact, silencing RIP140 seems to cause a slight extension in their spines. These results show that silencing RIP140 can be beneficial to neuron plasticity when cells are under stress. Reducing RIP140 level protects neurons from stress-induced damage, likely by relieving RIP140-mediated suppression of HSR.

**Fig. 4:**
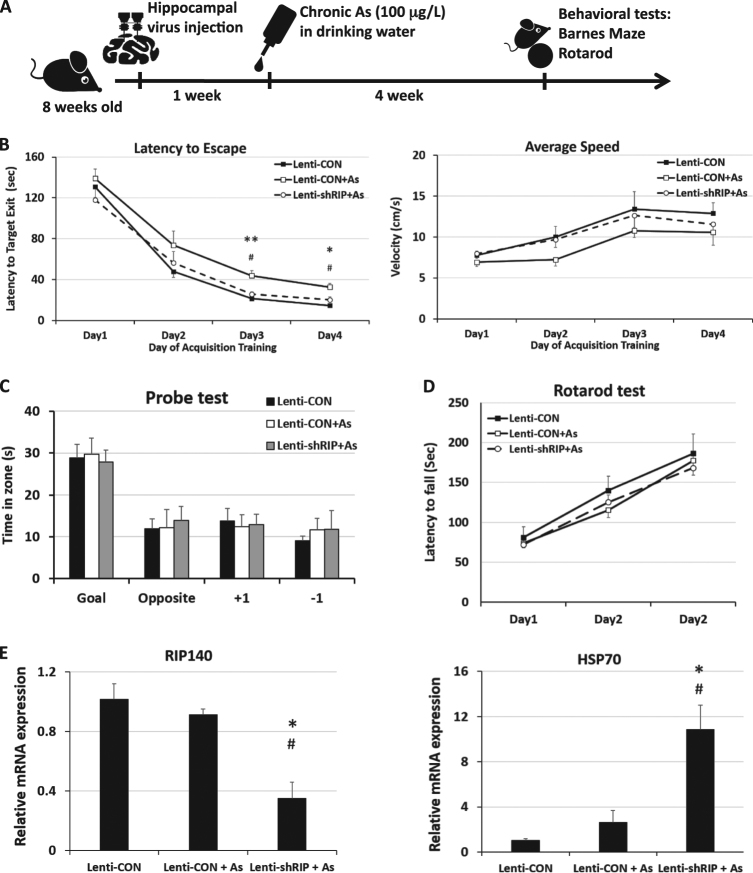
Silencing hippocampal RIP140 rescues As-suppressed hippocampus-dependent learning ability **a** Adult male mice injected, intra-hippocampally, with lentivirus (empty vector or shRRIP140) were allowed to recover for 1 week, and received As 100 μg/L in drinking water for 4 weeks. Mice performance in the Barnes maze test and then rotarod test are monitored. **b** Chronic As treatment affects learning performance, but not travel speed monitored in Barnes maze test (Lenti-CON, *N* = 10; Lenti-CON + As, *N* = 11; Lenti-shRIP+ As, *N* = 11). The deficit is rescued by silencing hippocampal RIP140. **c** Chronic As treatment or RIP140 knockdown has no significant effect on memory function monitored in Barnes maze test. **d** Chronic As treatment or RIP140 knockdown has no significant effect on motor skill learning monitored in rotarod test (Lenti-CON, *N* = 8; Lenti-CON + As, *N* = 11; Lenti-shRIP + As, *N* = 10). **e** Effects of chronic As treatment or RIP140 knockdown on hippocampal RIP140 and HSP70 mRNA expression (*N* = 4). Results are presented as means ± SEM, *p < 0.05, **p < 0.01 comparing Lenti-CON and Lenti-CON + As, ^#^ *p* < 0.05 comparing Lenti-CON + As and Lenti-shRIP + As. Statistical analyses performed by two-way repeated measure ANOVA with Bonferroni’s post hoc test

To further validate the functional role of RIP140 in protecting neurons from As-induced oxidative stress in vivo, we carried out chronic As treatment in mice with 100 μg/L As provided in drinking-water for more than 4 weeks^20^ (Fig. 4a). Before As treatment, 7–8-weeks-old mice were first administered, stereotaxically at hippocampal CA1 region, with lentiviruses carrying short hairpin RNA against RIP140 to silence endogenous RIP140 expression in the hippocampal CA1,^38^ or a control virus preparation. Animals were then assessed by Barnes maze test to determine hippocampus-dependent spatial learning and memory. The results show that chronic As exposure causes significant defects in animals’ learning as predicted. Interestingly, learning deficit in As-treated mice is rescued efficiently by first silencing RIP140 in their hippocampus (Fig. 4b, left). Importantly, there is no difference in animals’ mobility, as indicated by their average speed of movement (Fig. 4b, right), or spatial memory function (Fig. 4c) among the three groups. A 3-day rotarod test, used to motor function and evaluate hippocampus-independent motor skill learning shows no difference among the three groups (Fig. 4d).

Altogether these data show that lowering hippocampal RIP140 level is protective, it helps to maintain neuronal plasticity and hippocampus-dependent learning under stressful conditions. Lowering RIP140 level in a stressful situation provides a protective mechanism, relieving the otherwise suppressed HSR in order for animals to survive over stress such as chronic As toxicity.

### RIP140 is a co-repressor of HSF1

**Fig. 5:**
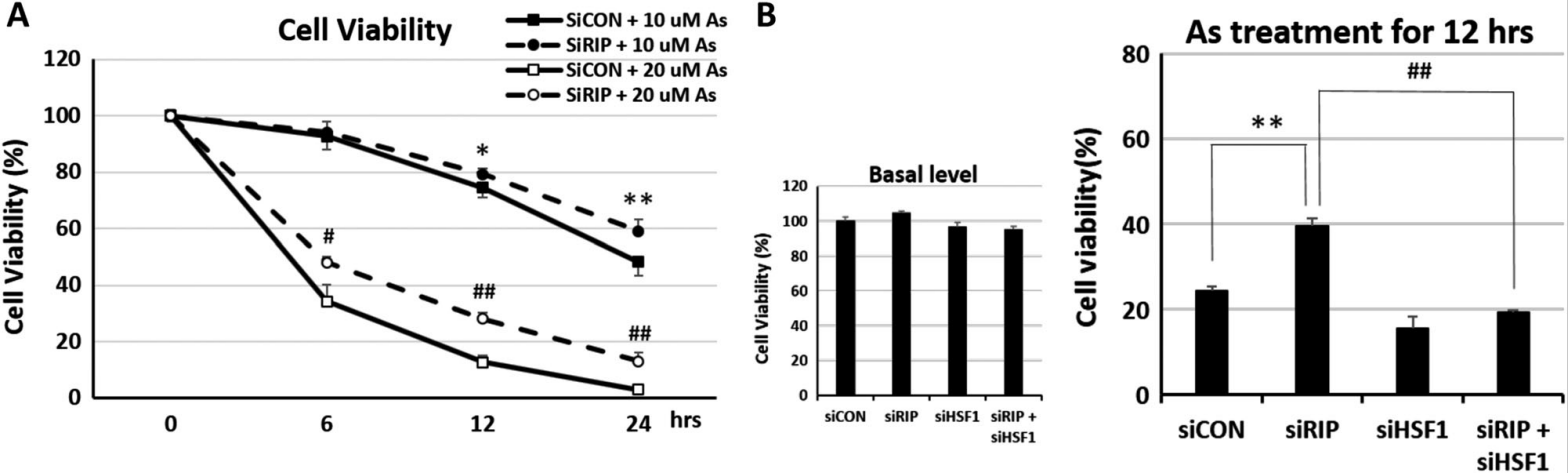
Silencing RIP140 protects neurons from As stress **a** MTT assay showing rescue of 10 and 20 μM As-induced cell death by RIP140 knockdown. *N* = 6 with two repeated tests, *p < 0.05, **p < 0.01 comparing siCON + 10 μM As and siRIP + 10 μM As groups; ^#^ *p* < 0.05, ^##^ *p* < 0.01 comparing siCON + 20 μM As and siRIP + 20 μM As groups. **b** RIP140 knockdown rescued cell survival is blocked by HIF1 knockdown. Results are presented as means ± SEM, *N* = 6 with two repeated tests, **p < 0.01, ^##^ *p* < 0.01 comparing the respective groups

To confirm if RIP140 modulates neuronal stress response by acting to repress HSF1, we monitored the effects of silencing RIP140 in HT22 cells exposed to 10 or 20 μM As (mild stress without reducing RIP140 protein level). As shown in Fig. 5a, As exposure decreases neuron viability as predicted, which can be rescued by silencing RIP140. Importantly, the rescuing effect of silencing RIP140 (to enhance HSR) is abrogated by silencing HSF1 (Fig. 5b, right), supporting the notion that RIP140 acts on HSF1 to modulate HSR. For controls, in unstressed cultures, neither the control, nor the RIP140 or HSF1 single knockdown affects neuron growth (Fig. 5b, left).

**Fig. 6:**
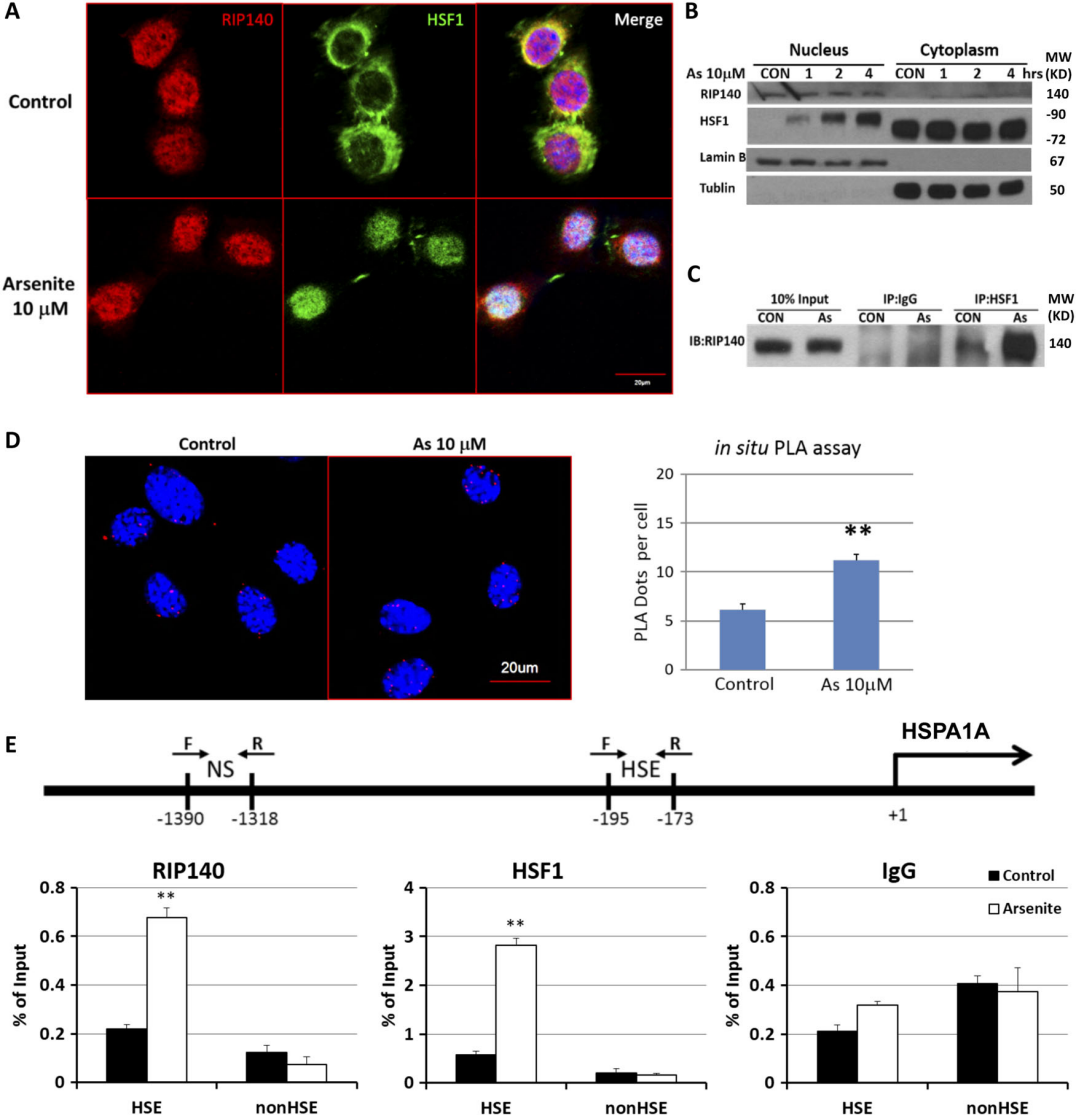
RIP140 attenuates HSR via association with HSF1 **a**, **b** HSF1 translocation to the nucleus after 1 h As treatment in HT22 cells. **c** Immunoprecipitation showing RIP140 associated with HSF1 in HT22 after As treatment. **d** In situ proximal ligation assay showing increased association of RIP140 with HSF1 after As treatment (*N* = 10/group). **e** Diagram showing heat shock element (HSE) and non-sense segment (NS) on the promoter of iHSP70. ChIP showing enhanced chromatin binding of RIP140 and HSF1 after As treatment (*N* = 3 with two repeated tests). Results are presented as means ± SEM, **p < 0.01 compared with the control group by independent *t*-test

To examine endogenous RIP140’s response and possible action in HSF1-mediated HSR, we monitored the localization of endogenous RIP140 and HSF1 in HT22 cells. Confocal images (Fig. 6a) and immunoblot data (Fig. 6b) show that RIP140 is predominantly distributed in the nuclei in unstressed neurons. In As-stressed neurons HSF1 is translocated from the cytosol to the nuclei as predicted; importantly, their signals overlap with anti-RIP140 signals in the nuclei only in stressed cultures, as shown in Fig. 6c. Moreover, in situ proximal ligation, used to monitor the proximity (indicating molecular interaction) of endogenous RIP140 and HSF1, shows a significant increase in co-localization of endogenous HSF1 with RIP140 following As stress (Fig. 6d). To determine if RIP140 is recruited to the expected chromatin targets, i.e., HSF1 target genes where HSEs are present, we employed CHIP to detect their binding to the HSE of iHSP70 (*Hspa1a*) promoter. Figure 6e shows robust recruitment of both HSF1 and RIP140 to the HSE of iHSP70 promoter. For a control, neither HSF1 nor RIP140 is detected on the non-specific (NS) chromatin region of this promoter. These data show that RIP140 acts as a co-repressor of HSF1 by binding, together with HSF1, to the HSE of HSF1 target genes.

### As stress induces RIP140 degradation, regulated by Syk-mediated tyrosine phosphorylation of RIP140

**Fig. 7:**
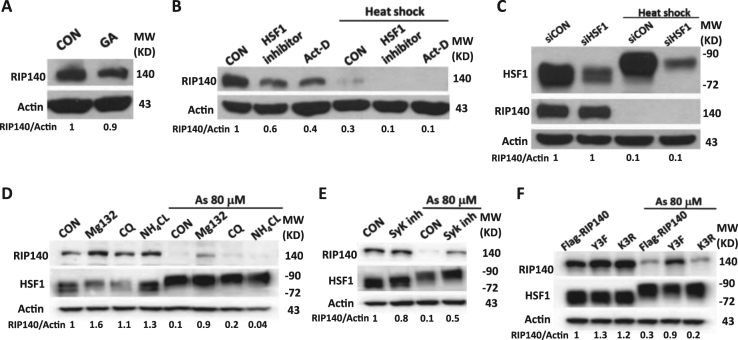
As-induced RIP140 degradation is triggered by Syk-mediated Tyr-phosphorylation **a** Immunoblot showing that HSF1 agonist GA fails to induce RIP140 degradation. **b** Immunoblot showing that HSF1 inhibitor KRIBB11 and transcription inhibitor Act-D both fail to block RIP140 degradation. **c** Immunoblot showing that HSF1 knockdown fails to block RIP140 degradation. **d** Immunoblot showing that the proteasome inhibitor (MG132), but not autophagy inhibitor (CQ and NH_4_Cl), attenuates RIP140 degradation. **e** Immunoblot showing that Syk inhibitor KN-62 attenuates As-induced RIP140 degradation. **f** Immunoblot showing Syk targeting sites-mutated RI140 (Y3F, Tyr 364, 418, 436) is not degraded following As stress. K3R is a negative control with substitution of Lys 662, 678, and 686 with Arg. Quantified and normalized expression level of RIP140 is shown under the corresponding blots

As the timing of RIP140 degradation parallels the timing of HSF1 activation (phosphorylation) and iHSP70 mRNA elevation after HS (Fig. 1a), an important question is whether HSF1 activation or HSR induces RIP140 protein degradation. Interestingly, in the presence of HSF1 agonist geldanamycin (GA), HSF inhibitor (KRIBB11), HSF-1-specific siRNA, or transcription inhibitor actinomycin D (Act-D), RIP140 does not disappear from the system (Fig. 7a, b, c). These results show that the disappearance of RIP140 is not caused by changes in HSF-1 status, or transcription regulation. Interestingly, proteosome inhibitor MG132, but not autophagy inhibitors, chloroquine (CQ), and NH_4_Cl, attenuates As-triggered disappearance of RIP140 (Fig. 7d), indicating RIP140 disappears from stressed neurons due to proteosome-mediated protein degradation. Since RIP140 can undergo extensive post-translational modifications^32,33,42^, mostly initiated by Ser/Thr- or Tyr-mediated protein phosphorylation, we then tested several kinase inhibitors, including spleen tyrosine kinase (Syk) inhibitor, CaMKII inhibitor KN-93, and MAPK kinase inhibitor U0126. We found that Syk inhibitor, but not CaMKII kinase or MAPK kinase inhibitor, attenuates As-triggered RIP140 degradation (Fig. 7e, Supplementary Fig. 2). This result suggests that Tyr-phosphorylation is involved. We tested several Tyr-mutated RIP140 mutants that may be regulated by ubiquitin-stimulated protein degradation, and found that a triple Tyr mutant (Tyr364, Tyr418, and Tyr436) (Y3F) is not degraded in As-stressed cells, whereas both the wild-type and a negative control (K3R, substitution of lysine with arginine at positions 662, 678, and 686) are degraded (Fig. 7f). These data show that As activates Syk to phosphorylate RIP140 at three-specific Tyr residues (positions 364, 418, and 436), which stimulates its subsequent ubiquitination and degradation by proteasome.

## Discussion

**Fig. 8:**
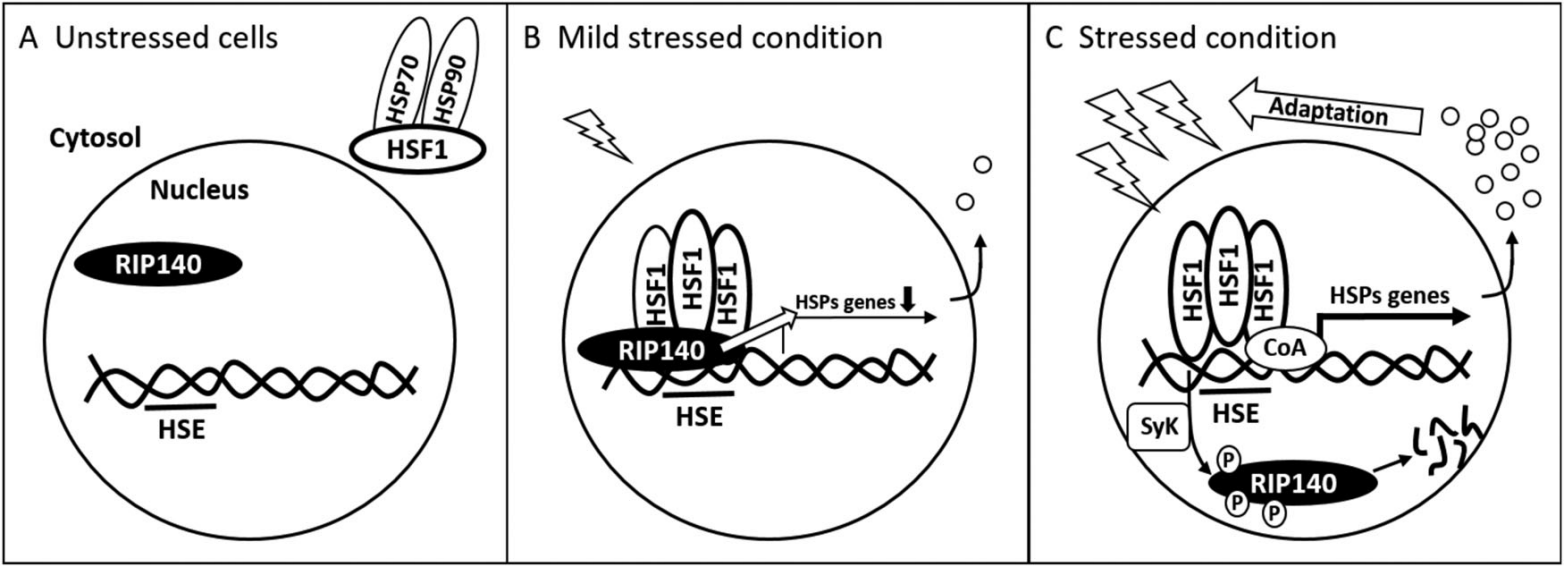
A scheme showing RIP140 modulating HSR (by co-repressing HSF1) under different stress conditions **a** In unstressed cells, HSF1, localized in the cytosol, is unable to activate its target genes. **b** During mild stress, HSF1 translocates to the nucleus to bind HSE and activate its chromatin targets; but RIP140 associates with HSF1 to suppress/dampen HSF1’s transcriptional activity. **c** In stressed cells, Syk is activated to phosphorylate RIP140 for its degradation; the suppression of HSF1 is thus relieved, enabling a full scale HSR that facilitates adaptation and recovery

The current study reveals a neuron-protective mechanism that exploits rapid degradation of RIP140 to modulate neuron’s HSR under stressful conditions such as HS or As exposure (Fig. 8). During normal, or a mild stressful condition, RIP140 acts as a co-repressor of HSF1 to suppress genes that are otherwise activated in HSR. During stressful conditions, RIP140 is rapidly lost due to Tyr phosphorylaton-stimulated proteosome-mediated protein degradation, thereby enabling cells to timely activate HSF-1 target genes to execute HSR. We provide both In vitro and In vivo data to demonstrate that lowering RIP140 level protects neurons and animals from As-induced oxidative stress. We also demonstrate that RIP140 is recruited, together with HSF-1, to the target genes of HSF-1 during HSR, and that phosphorylation on three-specific Tyr residues is critical to RIP140’s degradation, which is mediated by Syk.

An immediate question is, why HSR should be suppressed in unstressed cells. Presumably, undesired elevation of HSR in unstressed cells could disrupt normal proteostasis. Genes activated during HSR could affect protein biosynthesis, folding, degradation, and trafficking, which are needed only during a stressful condition. Alternatively or additionally, HSF1 can function as an oncoprotein,^43^ and HSF1‐mediated HSR contributes to stress resistance, which can be hijacked by malignant cells to promote oncogenesis.^43,44^ A large body of literature has provided evidence for constitutive HSF-1 activation in cancer cell growth, invasion, and metastasis.^44,45^ In the mouse, *Hsf1* knockout reduces Trp53 deficiency-triggered tumorigenesis and chemically induced skin carcinogenesis.^43,46^ Thus, inactivating or dampening HSFR may minimize the oncogenic potential of normal cells. To this end, RIP140 can suppress undesired HSF-1 activation to minimize the possibility of unnecessary HSR. In the future it would be interesting to examine whether RIP140 is lost in cancers.

Our behavioral results show that silencing RIP140 in hippocampus rescued chronic As-induced learning deficits. However, it has been reported that RIP140 knockout mice exhibited impaired hippocampal-dependent learning and memory in Morris water maze test,^47^ suggesting that RIP140 itself, under a normal condition, could be beneficial to neural plasticity, and learning/memory. Indeed, silencing RIP140 alter the spine morphology and increases the ratio of spine with mushroom shape in primary hippocampal neuron cultures (Fig. 3). It may be interesting to study the role of RIP140 in spine plasticity also by overexpressing RIP140 in the future. In this current study, our results reveal the benefit of reducing RIP140 level only in the context of stress induction, such as upon exposure to As. Without stress, RIP140 itself is important, which may involve its actions on many other genes as it is a wide-spectrum co-regulator of many transcription factors.^48–50^

When neurons are stressed RIP140 is rapidly degraded, which provides a timely mechanism enabling efficient HSF1 activation. Interestingly, our data reveal that RIP140 degradation is mediated by proteosome and involves three Tyr residues phosphorylated by Syk (Fig. 7). This indicates that the Syk may also act as a stress sensor. Indeed, the enzyme activity of Syk can be induced by oxidative stress, ER stress, inflammatory stress, and osmotic stress.^30,51–53^ In B cells, H_2_O_2_-induced Syk activation is involved in protecting cells from apoptosis and G2/M arrest.^51^ In microglia, As (250 μM) and proteosome inhibitor MG132 induce stress granule formation and Syk activation, which contributes to the clearance of stress granules.^52^ Chronic exposure to Aβ or As (1 μM) also induces ROS production, stress granule formation and Syk activation.^53^ These studies support our findings, and are consistent with the notion that stresses-induced Syk activation results in RIP140 degradation, which contributes to rapid HSR.

Many neurodegenerative diseases involve protein misfolding and/or aggregation, such as Aβ and tau in AD, α-synuclein in Parkinson’s disease, Huntingtin in Huntington’ disease, and superoxide dismutase in Amyotrophic lateral sclerosis.^3,54^ The primary risk factor for developing neurodegenerative diseases is aging with accumulation of oxidative stress and proteotoxicity. To this end, HSR plays an important role in modulating these stress responses.^1,55,56^ HSF1 and HSPs (molecular chaperones), especially HSP70, have been proposed as potential therapeutics for these diseases, and demonstrated in neurodegenerative disease models.^57–59^ Interestingly, RIP140 is enriched in cortex and hippocampus, areas associated with AD pathology. It is tempting to speculate a potential role for RIP140 in the progression/development of these diseases. This awaits further studies of animal models and human patients. Further, targeting RIP140 protein quality control may provide a potential preventive or therapeutic strategy for neurodegenerative diseases.

## Materials and methods

### Reagents

Sodium arsenite (S7400), chloroquine diphosphate salt (C6628), and ammonium chloride (A9434) purchased from Sigma were dissolved in ddH_2_O. actinomycin D (A1410), MG132 (474787) and geldanamycin (G3381) purchased from Sigma were dissolved in dimethyl sulfoxid (DMSO). HSF1 inhibitor KRIBB11 purchased from Medchemexpress (HY-100872) was dissolved in DMSO.

### Cell culture

HT22 cells (from Salk Institute) were maintained with Dulbecco’s modified eagles medium (Gibco) supplemented with 10% fetal bovine serum (Gibco), penicillin and streptomycin. Primary hippocampal neurons were isolated as described.^38^ Briefly, hippocampus from embryonic (E18.5) mouse brain was digested in 0.25% trypsin, and cultured on poly-d-lysine-coated plates (5 lg/mL) in neurobasal medium supplemented with B27 at 37 °C under 5% CO_2_. The experiments were carried after 11–14 days of culture (DIV 11–14).

### Quantitative real-time PCR (qPCR)

Total RNA was isolated using TRIzol (Invitrogen), cDNA was synthesized using Omniscript RT kit (QIAGEN), and qPCR was performed using SYBR-Green (Agilent) and detected with Mx3005 P (Agilent). The primers are: iHSP70, forward 5′- TGG TGC TGA CGA AGA TGA AG-3′, reverse 5′- AGG TCG AAG ATG AGC ACG TT-3′; Actin, forward 5′- CAA CGG CTC CGG CAT GTG-C-3′, reverse 5′- CTC TTG CTC TGG GCC TCG-3′; HSP90, forward 5′- **AGC TTT CAG AGC TGT TGC GGT-**3′, reverse 5′- TTG CTG TCC ACC CAT ATG TGC-3′; HSP40, forward 5′-TTC GAC CGC TAT GGA GAG GAA-3′, reverse 5′- CAC CGA AGA ACT CAG CAA ACA-3′; HSP27, forward 5′-CGG GCC TCG AAA GTG ACC GG-3′, reverse 5′-TTC GAC CGC TAT GGA GAG GAA-3′; HSF1, forward 5′- TGG TGC TGA CGA AGA TGA AG-3′, reverse 5′- AGG TCG AAG ATG AGC ACG TT-3′.

### Western blotting and immunoprecipitation

Western blotting was conducted as described^60^ using Anti-β-actin (SC-47778, Santa Cruz), Anti-RIP140 (9101, Cellular Signaling) and Anti-HSF1 (SC-17757, Santa Cruz). For immunoprecipitation, 500 μg whole-cell lysate incubated with 5 μg antibodies for 2–3 h in Co-IP buffer (50 mM Tris–HCl pH 8.0, 10% glycerol, 100 mM NaCl, 1 mM EDTA and 0.1% NP-40) was incubated with protein G beads (Upstate) overnight. After centrifugation, beads were washed using Co-IP buffer, and the precipitates were subjected into SDS-PAGE for western blotting.

### In situ proximal ligation assay

In situ proximal ligation assay was performed using Duolink PLA assay kit (Olink Bioscience) according to the manufactural instruction. In brief, 4% polyparaformaldehyde-fixed cells were incubated with primary antibodies overnight at 4 °C. To detect the endogenous complex of RIP140 and HSF1, anti-RIP140 (ab42126; Abcam; 1:200) and anti-HSF1 (sc-17757; Santa Cruz; 1:400) were used. Images were acquired under Olympus FluoView 1000 IX2 upright confocal microscope.

### Cell viability (MTT Assay)

HT22 plated at equal density was solubilized with DMSO at indicated time points of As treatments after MTT (3-[4,5-dimethylthiazol-2-yl]-2,5-diphenyltetrazolium bromide; thiazolyl blue) incubation for 4 h. Absorbance was measured at 570 nm.

### Animal experiments

To avoid potential complication from the effects of menstrual variation on behavioral performances, we utilized only male animals in these experiments. Six-weeks-old C56BL/6J mice from Jackson Laboratory were maintained in the animal facility of the University of Minnesota, in a temperature controlled room (22 ± 1 °C) on a 14/10 light dark cycle (lights on/off at 0600/2000) with ad-lib food and water. Experimental procedures were conducted according to NIH guidelines and approved by the University of Minnesota Institutional Animal Care and Use Committee. All efforts were made to reduced animals’ suffering and the number of animals used.

### Stereotaxic injection

Seven to eight-weeks-old mice were anesthetized with ketamine mixed with Xylazine hydrochloride injection (100/10 mg/kg, respectively) and placed on a stereotaxic instrument (Stoelting, Wood Dale, IL). Two microliters of lentiviral vectors were delivered bilaterally to hippocampus at anteroposterior 2.0 mm, medial-lateral 1.2 mm and dorsoventral 1.6 mm as previously described^40^. Seven days after surgery, mice received chronic As treatment for 3 weeks and then subjected to behavioral analyses. Mice are randomly assigned to each experimental groups (Lenti-CON; Lenti-CON + As; Lenti-shRIP + As).

### Behavioral assessments

Twenty-six male mice were used in Barnes maze and rotarod test (Lenti-CON, *n* = 10; Lenti-CON + As, *n* = 11; Lenti-shRIP + As, *n* = 11). All behavioral assessments were performed during the light phase following habituation to the test room for 1 h each day.

The Barnes maze, a dry-land maze test for spatial learning and memory, was modified from a previous procedure.^61^ The maze consists of a circular platform with 20 holes along the perimeter (Med-Associated, St. Albans, Vermont). During training sessions, only one hole is open. Animals receive reinforcement to escape from the open platform surface to a small, dark chamber located under one the holes called the “target box”. The circular platform was divided into four zones, including goal quadrant with the escape hole, +1, −1, and the opposite quadrant. Each trial started by placing the animal in a white cylinder at the center of the platform, which was removed after 15 s. Mice underwent acquisition of spatial learning for four consecutive days and four trials per day with visible cues. On Day 5, spatial reference memory was evaluated by a 90 s probe test when all the holes were closed. Mice freely explored the maze and visited the target hole and the adjacent holes. Latency to reach the target hole for the first time, travel distance and time spent in each quadrant were recorded and analyzed by EthoVision XT (Noldus).

Rotarod, a standard test of motor coordination and balance in rodents, was modified from a previous procedure.^62^ The rotarod apparatus (Ugo Basile) consists of a rotating cylinder (approximately 3 cm in diameter at an increased speed ranging from 4 to 40 rpm). The animal was placed on the cylinder, moved continuously to stay from falling. Latency at which mice fall off the rotating cylinder was measured. Maximal trial length is 300 s. Rotarod performance was assessed three times daily for three consecutive days. The multiple measures provide an assessment of motor skill learning.

### Statistical analyses

Statistical differences between groups were determined by two-way or two-way repeated measure analysis of variance (ANOVA) followed by Bonferroni’s post hoc test. Independent-sample *t*-tests were used to compare two independent groups. Statistical analyses were performed by SPSS 17.0. All tests were performed at a significance level of *p* < 0.05, and data were presented as the mean ± SEM.

## Electronic supplementary material

Supplement
