# Supplementary material for: Receptor-interacting protein 140 as a co-repressor of Heat Shock Factor 1 regulates neuronal stress response
Source: Cell Death Dis. 2017 Dec 12;8(12):3203. doi: 10.1038/s41419-017-0008-5 (PMC5870597; doi:10.1038/s41419-017-0008-5)
Supplement: Supplementary file 1 — Supplement [file 41419_2017_8_MOESM1_ESM.pdf]

Supplementary data

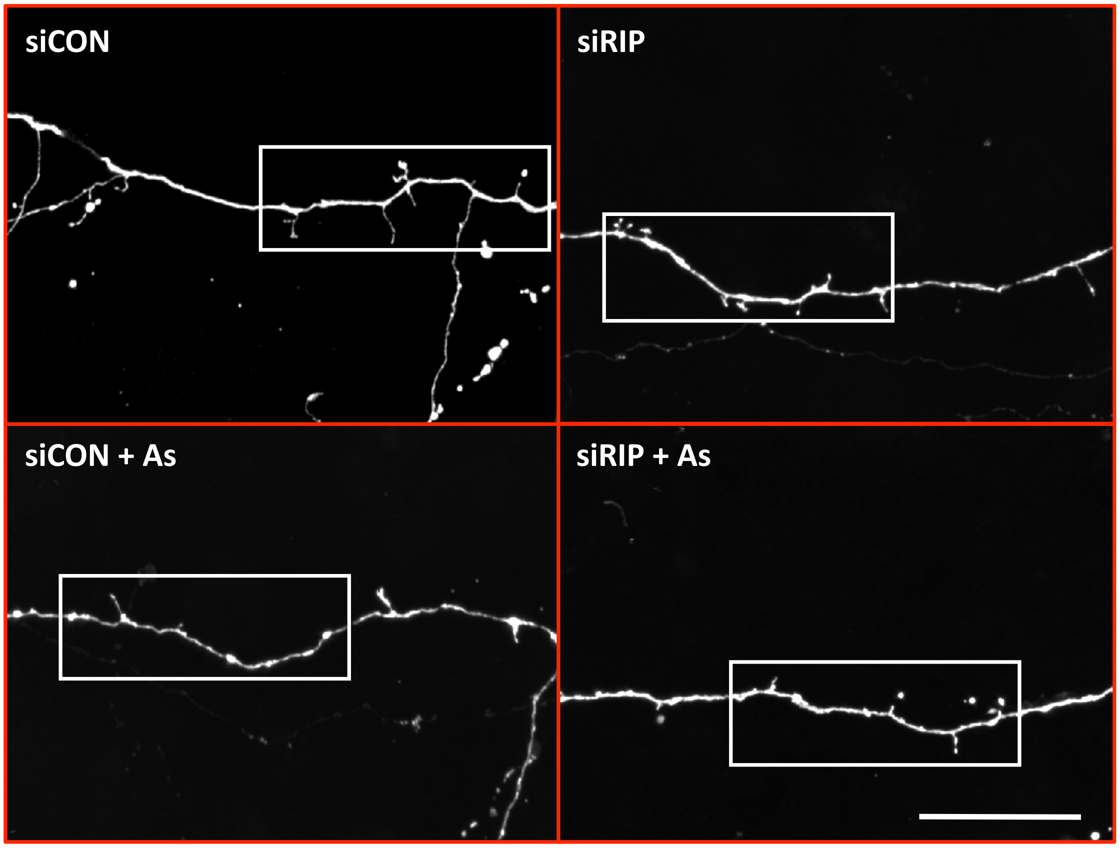

Supplementary Figure 1. Silencing RIP140 rescue As-reduced spine density.
Images of the boxed areas are shown in Fig 3B left. Scale bar = 10 μM.

Supplementary data

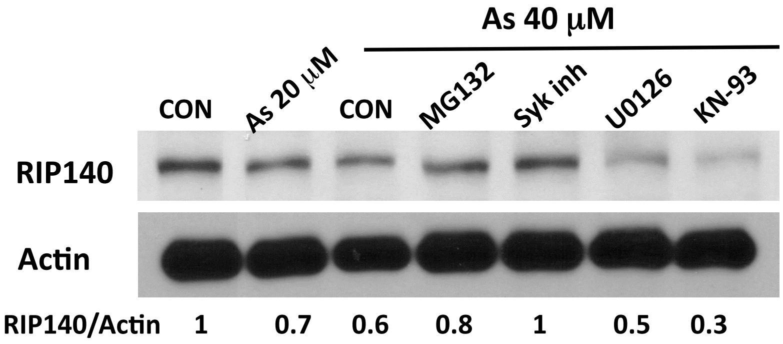

Supplementary Figure 2. MG132 and Syk inhibitor block As-induced RIP140 degradation

Immunoblot showing that proteinase inhibitor MG132 and Syk inhibitor block As-induced RIP140 degradation, but not MEK kinase inhibitor U0126 and CaMKII inhibitor KN93. Quantified and normalized expression levels of RIP140 are shown under the corresponding blots
